# A comparison of isometric and isokinetic normalization methods for electromyographic data from sub-regions of supraspinatus and infraspinatus during dynamic tasks

**DOI:** 10.1080/23335432.2023.2210634

**Published:** 2023-05-14

**Authors:** Ronelle Calver, Alan Cudlip, Clark R. Dickerson, Prosanta Mondal, Scotty Butcher, Soo Y. Kim

**Affiliations:** a Department of Medicine, Physical Medicine and Rehabilitation, McMaster University, Hamilton, Canada; b College of Medicine, Department of Physical Medicine and Rehabilitation, University of Saskatchewan, Saskatoon, Canada; c College of Medicine, School of Rehabilitation Science, University of Saskatchewan, Saskatoon, Canada; d Department of Kinesiology, Neuromechanics and Ergonomics Lab, Brock University, Ontario Canada; e Faculty of Applied Health Sciences, Department of Kinesiology, Digital Industrial Ergonomics and Shoulder Evaluation Laboratory (DIESEL), University of Waterloo, Ontario, Canada; f College of Medicine, Department of Community Health and Epidemiology, University of Saskatchewan, Saskatoon, Canada

**Keywords:** Supraspinatus sub-regions, infraspinatus sub-regions, fine-wire EMG, normalization

## Abstract

This study explored effects of using isometric versus isokinetic maximal voluntary contractions (MVCs) to normalize EMG data from supraspinatus and infraspinatus subregions during isokinetic tasks. Participants performed submaximal isokinetic external rotation (ER) and scaption tasks at two speeds. Three isometric MVCs were used: seated ER; side-lying scaption; side-lying abduction. Isokinetic MVCs were performed in the same position and speeds as the experimental tasks. Data were normalized using peak EMG from reference tasks: MVC which produced the greatest amplitude overall (MEA), isometric MVC with greatest amplitude (isometric best), isokinetic MVC with greatest amplitude (isokinetic best), and the greatest amplitude from the isokinetic MVC that matched the experimental task (isokinetic matched). Mean %MVC from each experimental task/ sub-region were compared by normalization method. The isokinetic matched method versus the MEA method was significantly different in all comparisons with isokinetic matched resulting in relative normalized task values up to 162% greater. The isometric best method resulted in significantly greater %MVC 37% of the time compared to the MEA method, whereas there were no differences when using isokinetic best compared to MEA. Isokinetic MVCs are less likely to overestimate %MVC than isometric and their use should be considered when normalizing data from dynamic tasks.

## 1. Introduction

Electromyography (EMG) provides insight into muscle function, but in order to compare across participants and between studies, normalization of EMG data is necessary (De Luca 1997; Lehman and McGill 1999). At present, no clear consensus exists on the most appropriate method of normalization (Burden 2010; Halaki and Ginn 2012). A common method used to normalize these data is to divide the EMG from the experimental task by the peak amplitude obtained during a maximal effort reference task. The EMG data from the experimental task is thus represented as a percentage of maximal activity (Burden 2010; Halaki and Ginn 2012). Whether this reference task should be isometric or isokinetic when the experimental task involves a dynamic movement, particularly those performed at high velocity, is equivocal (Ball and Scurr 2013). Previous studies with high velocity dynamic tasks, for example swimming and fastball pitching, were normalized to isometric maximal voluntary contractions (MVCs) have resulted in values greater than 100% for the experimental task, which should be, by definition, impossible if full activation of the muscle is achieved during the MVC (Clarys et al. 1983; Jobe et al. 1984). If an isometric MVC does not elicit maximal muscle activity and it is then used to normalize a dynamic task, the resulting normalized %MVC values are subsequently inflated and in a clinical or occupational setting this may result in tasks being interpreted as potentially unsafe due to high levels of muscle activation (Fischer et al. 2010). Additionally, the development of new EMG-driven biomechanical models rely on the magnitude of the inputted EMG data to provide accurate outcomes (Dickerson et al. 2008). If the EMG data is not an accurate representation of the true activation of the muscle in question, the product of the models may suffer. In a review by Ball & Scurr they suggest the reference task used for normalization should be similar to the dynamic task being investigated, thus promoting similar neural input and motor unit recruitment (Ball and Scurr 2013). With the use of an isometric MVC for normalization, the shorter time to fatigue fast twitch muscle fibres and the effect of rapid recruitment and de-recruitment of motor units during a dynamic task may not be accounted for, which may alter the interpretation of experimental task data (Ball and Scurr 2013). By using a normalization task similar to the experimental task, these effects might be better accounted for, but there is limited research assessing such methods.

There are few studies that have compared the effect that isometric versus isokinetic normalization methods have on experimental task EMG data. Hodder & Keir reported that maximal electrical activity of various shoulder muscles most often occurred during isokinetic MVCs and there were significant differences when using isometric versus isokinetic MVCs to normalize data from a clockwise and counterclockwise wheel-spinning task performed at 60°/sec and 120°/sec (Hodder and Keir 2013). The isokinetic MVCs in this study were performed at 30°/sec using a dynamometer and were flexion/extension from 0–90° elevation, abduction/adduction from 0–90° elevation, and bent elbow ER/IR with the humerus abducted to 45° performed from 0° to the maximum comfortable external rotation (Hodder and Keir 2013). This study considered each muscle as a single entity, but recent research has identified anatomical sub-regions in some muscles of the rotator cuff, including anterior and posterior sub-regions within supraspinatus (Roh et al. 2000; Kim et al. 2007) and superior, middle, and inferior sub-regions within infraspinatus (Fabrizio and Clemente 2014; Bacle et al. 2017). EMG studies using intramuscular fine-wire electrodes to specifically target these sub-regions have demonstrated functional differences (Kim et al. 2017; Cudlip and Dickerson 2018; Alenabi et al. 2019; Zaluski et al. 2021; Calver et al. 2022). Previous research examining dynamic free weights tasks normalized to both isometric and isokinetic MVCs found significant differences when comparing the two forms of normalization for specific tasks for these sub-regions (Calver et al. 2022). To further investigate the difference in function of these sub-regions, including the impact exercise has on force applied to different parts of the tendon, appropriate normalization method needs to be consistently employed to allow accurate comparison and interpretation of data. The aim of this study was to investigate what differences in data interpretation occur when using current standard isometric versus isokinetic MVC tasks performed at two speeds (30°/sec and 90°/sec) to analyze EMG data from supraspinatus and infraspinatus sub-regions during dynamic tasks. We hypothesized isokinetic MVCs at both angular speeds would result in greater amplitudes in the majority of participants when compared to isometric MVCs allowing for normalized data to be more reliably be placed on a 0–100% scale.

## 2. Methods

### 2.1. Participants

Sixteen healthy participants (mean age 32 yrs (23–58 yrs), height 170 cm (112-175 cm), weight 71.7 kg (75-81 kg), 4 M/12F) were recruited and informed consent obtained prior to testing. Exclusion criteria were previous shoulder injury or known pathology, neuromuscular conditions, implanted devices, cardiovascular conditions, bleeding disorders, and use of anti-coagulant medications. The local Biomedical Research Ethics Board approved this study. Participants attended two sessions; in the first session they were familiarized with the dynamometer and the equipment was adjusted to fit each subject with these settings recorded and in the second session intramuscular electrodes were placed followed by MVC and task data collection.

### 2.2. Intramuscular electromyography

Intramuscular electrode insertion was performed by a single researcher and followed previously published protocols for supraspinatus (Kim et al. 2017) and infraspinatus (Alenabi et al. 2018; Kim et al. 2019) sub-regions. Given the target depth for anterior and posterior supraspinatus, custom-made bipolar electrodes were required and ultrasound guidance (12 MHz linear array transducer; GE Logic E, GE Medical Systems, Milwaukee, Wisconsin, USA) was used to confirm placement. These custom electrodes were inserted using 90 mm/23 gauge hypodermic needles (Quinke Point, Kimberly-Clark Spinal QP Needle). Commercial sterile single use hypodermic 50 mm/25 gauge paired – fine wire needle electrodes (Product # 000-318-150, Motion Lab Systems, Inc., Baton Rouge, LA) were used for infraspinatus sub-regions. All wires were taped to the skin and connected to a Trigno™ spring contact sensor that sampled at 2000 Hz (Delsys®, Inc. MA, USA).

### 2.3. Maximal voluntary contractions

#### 2.3.1. Isometric maximal voluntary contractions

**Figure 1.**
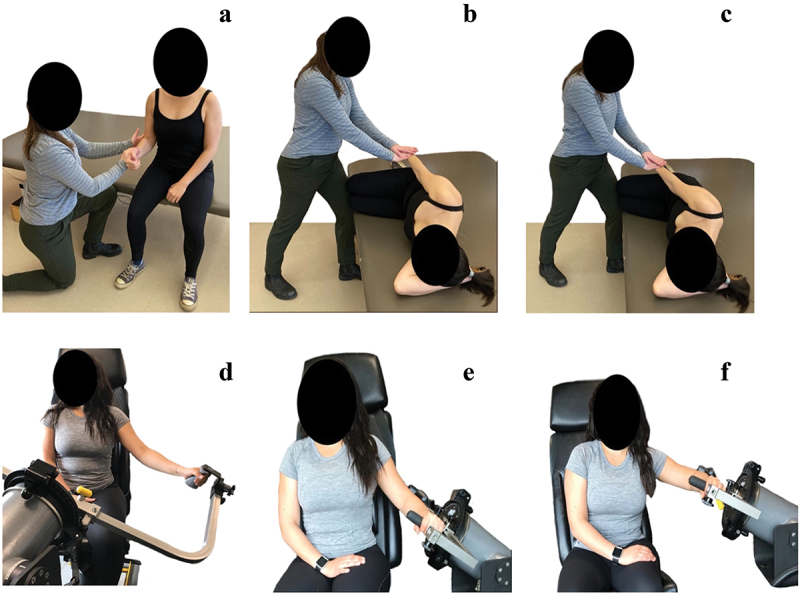
Visual representation of isometric and isokinetic MVCs: isometric ER (a), isometric abduction (b), isometric scaption (c), isokinetic scaption (d), isokinetic ER30° (e), isokinetic ER90° (f). For isokinetic MVCs two speeds were used (30°/sec = slow, 90°/sec =fast), with the start position demonstrated within the figure. Full details regarding MVC postures may be found in Table 1.

Three standard isometric MVCs were performed: external rotation (isometric ER), abduction in the frontal plane (isometric abduction), and abduction in the scapular plane (isometric scaption). Isometric ER was performed in a seated posture and isometric abduction and scaption were performed in a side-lying posture. These postures were chosen as they are commonly used and accepted as the current standards to normalize EMG data from the rotator cuff (Boettcher et al. 2008; Waite et al. 2010; Hodder and Keir 2013; Kim et al. 2017) The participant was instructed to ramp up his or her effort over one second, hold at peak exertion for three seconds, and then to release over one second, for a total working time of 5 seconds (Chaffin 1975). This was done against resistance applied by a research assistant. Details of the isometric MVC postures can be found in Table 1 and Figure 1.

**Table 1.** Description of MVCs.

MVC Name	Description	Angular Speed
Isometric abduction	In side lying, the arm is held in 10° of abduction with the elbow extended and external force applied at the wrist.	N/A
Isometric scaption	In side lying, the arm is held in 10° of scaption with the elbow extended and external force applied at the wrist	N/A
Isometric ER	Seated, the arm is held close to the body with 90° of elbow flexion in neutral. External force is applied at the wrist while the participant attempts to externally rotate the arm.	N/A
Isokinetic scaption	Seated on the dynamometer, the elbow is extended and handle held at lowest comfortable position with the thumb pointed up (full can). The arm is then elevated to 110° in the scapular plane.	30°/sec, 90°/sec
Isokinetic ER30 °	Seated on the dynamometer, the elbow is bent to 90° and the shoulder held in 30° abduction. External rotation is then performed to maximum comfortable range.	30°/sec, 90°/sec
Isokinetic ER90°	Seated on the dynamometer, the elbow is bent to 90° and the shoulder held in 90° abduction. External rotation is then performed to maximum comfortable range.	30°/sec, 90°/sec

#### 2.3.2. Isokinetic maximal voluntary contractions

Using a Humac NORM dynamometer (CSMi, Stoughton, MA, USA), three isokinetic MVCs were performed at two angular speeds: fast (90°/sec) and slow (30°/sec). The isokinetic MVC positions were as follows: scaption (isokinetic scaption), external rotation with the arm abducted to 30° and elbow flexed to 90° (isokinetic ER30°), and external rotation with the arm abducted to 90° and elbow flexed to 90° (isokinetic ER90°). For external rotation MVCs, the participant started with their arm in a neutral forward position before externally rotating their arm to the greatest point at which they felt comfortable exerting force. For the scaption MVC, the participant started with their arm by their side and raised their arm to 110°. Details of the initial isokinetic MVC postures can be found in Table 1 and Figure 1.

### 2.4. Experimental task

The experimental tasks matched each of the isokinetic MVCs for both position and angular speeds (Table 1 and Figure 1) and were chosen as they will be used in a subsequent study investigating the activation of the sub-regions of supraspinatus and infraspinatus. The peak torque achieved by the participant during isokinetic MVC testing in each of these positions was recorded. A visual target set at 50% of each participant’s peak torque for a given task was then provided on a screen and they were instructed to perform five repetitions of the movement with the goal of matching their effort to this target. As the arm of the dynamometer moved, the participant could watch in real time how closely the force they were exerting matched the set target on the screen allowing them to adjust their effort as needed. The participant had an opportunity to practice matching their force production to the target for each task prior to data collection.

### 2.5. Experimental protocol

During the testing day, all intramuscular EMG electrodes were placed as indicated above. A 10-second minimum quiet trial was then recorded with the participant lying in prone. The isometric MVCs were then performed in randomized order. Prior to data collection, participants had an opportunity to practice exerting force in each of the MVC positions to ensure appropriate postures and comfort. For both isometric and isokinetic MVCs, two repetitions were performed with a minimum two minutes of rest between repetitions. To minimize the time required by the participant, an isokinetic MVC was performed followed by the five repetitions at 50% of their maximal torque in this same configuration. The order that the isokinetic MVC/task were performed was randomized first by type and then by angular speed. The randomized isokinetic MVC task immediately followed by the matched randomized task was performed in this manner as it took significant time to readjust the dynamometer for each change in settings. A minimum one-minute break separated each task performed.

### 2.6. Data analysis

EMG was analyzed with respect to amplitude. Raw EMG signals were high-pass filtered at 30 Hz to remove ECG contamination (Drake and Callaghan 2006), then full-wave rectified and linear enveloped using a single pass second-order low pass filter at 4 Hz; this cutoff is commonly used for the low frequency motion of upper extremity musculature (Cudlip et al. 2015; Cudlip and Dickerson 2018). Before normalization, trials were visually inspected and cropped prior to EMG onset of the first repetition and after EMG offset to remove excess data at the start and ends of each trial. Isometric and isokinetic MVCs were processed identically. Data were normalized against four MVC peak values in parallel. The values used for normalization were peak values in the muscle-specific MVC which produced the greatest amplitude overall (MEA), the isometric muscle-specific MVC with the greatest amplitude (isometric best), the isokinetic MVC with the greatest amplitude (isokinetic best), and the isokinetic MVC that matched the experimental task with respect to angular speed and postural range (isokinetic matched). All normalized values were represented as %MVC.

### 2.7. Statistical analysis

Due to inconsistent pick-up of EMG signal from the inferior infraspinatus, this sub-region was excluded from analysis. To compare means among four normalizing methods (MEA, isokinetic best, isometric best, and isokinetic matched) for each of the four sub-regions (anterior supraspinatus, posterior supraspinatus, superior infraspinatus, middle infraspinatus) and six tasks (isokinetic scaption fast, isokinetic scaption slow, isokinetic ER30° fast, isokinetic ER30°slow, isokinetic ER90° fast, isokinetic ER90°slow) ANOVAs for dependent/correlated samples were performed. Bonferroni’s adjusted p-value of 0.0083 was used for pairwise comparison (post-hoc analysis). The MVC that produced MEA in each individual subregion were also recorded (16 participants x 4 subregions = 64 individual subregions). See Table 2. All statistical analyses were conducted using SAS 9.4 (SAS Institute, Inc., Cary, USA).

**Table 2.** Number of participants with MEA found for each sub-region based on MVC position.

	Normalization Method										
	Isometric				Isokinetic						
Sub-region	Abduction	Scaption	ER	Total isometric	Scaption fast	Scaption slow	ER90° fast	ER90° slow	ER30° fast	ER30° slow	Total Isokinetic
Anterior supraspinatus	3	1	2	6	**5**	3	1	1	0	0	10
Posterior supraspinatus	**3**	**3**	1	7	2	**3**	1	2	1	0	9
Superior infraspinatus	**5**	2	3	10	2	1	0	2	0	1	6
Middle infraspinatus	2	2	2	6	1	0	2	**3**	1	**3**	10
Total (*n* = 64)	29				35						

## 3. Results

### 3.1. Maximum electrical activity by MVC

Both isokinetic and isometric MVCs produced MEA in each sub-region. Isokinetic MVCs produced MEA in 35 individual sub-regions versus 29 individual sub-regions from isometric MVCs (Table 2). Isokinetic MVCs were more likely to result in MEA for anterior and posterior supraspinatus sub-regions (10 and 9 individuals respectively) as well as for the middle infraspinatus sub-region (10 individuals) (Table 2). Abduction and scaption based MVCs more often resulted in MEA for anterior and posterior supraspinatus sub-regions (12 and 11 individuals respectively) and for the superior infraspinatus sub-region (10 individuals) (Table 3).

**Table 3.** Number of participants with MEA for each sub-region comparing grouped scaption/abduction MVC position versus grouped ER MVC position.

	MVC position	
Sub-region	Abduction & Scaption	ER
Anterior supraspinatus	12	4
Posterior supraspinatus	11	5
Superior infraspinatus	10	6
Middle infraspinatus	5	11
Combined sub-regions	38	26

### 3.2. Difference in mean %MVC using different methods of normalization

**Figure 2.**
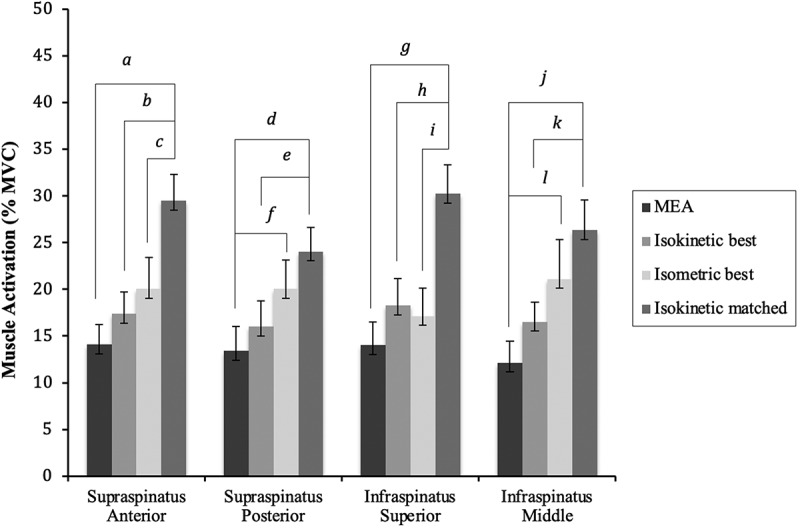
Graphical representation of the muscle activation of each sub-region during the isokinetic ER30° fast task with significant differences in %MVC between normalization methods marked with letters. Alpha level was adjusted to 0.0083 (0.05/6) using Bonferroni correction. Standard error of the mean is represented by bars with caps. a= *p* < 0.0001, b= *p* < 0.0001, c= p 0.0008, d= *p* < 0.0001, e= p 0.0003, f= p 0.0022, g= *p* < 0.0001, h= *p* < 0.0001, i= *p* < 0.0001, j=p < 0.0001, k= p 0.0032, l= p 0.0067.

Significant differences (p-value<0.0083) between normalization methods for each sub-region and experimental task occurred (Table 4). Normalizing to isokinetic matched resulted in higher %MVC values compared to normalizing data using the MEA method for all sub-regions in all tasks (*c*). The difference was greatest for the infraspinatus middle sub-region during the scaption fast task with a %MVC normalizing with MEA of 9.48% versus isokinetic matched at 24.87%, which is a relative difference of 162% (p-value<0.0001). Using the isokinetic matched method for normalization resulted in higher values than using the isometric best method for five out of the six experimental tasks for the superior infraspinatus sub-region with relative differences ranging from 42–93% (*f*) (p-values<0.0001–0.0069). Using the isokinetic matched method resulted in significantly higher %MVC than using the isokinetic best method for both supraspinatus sub-regions and the superior infraspinatus in all four ER tasks (relative difference 47%-69%, p-values<0.0001–0.0044) as well as for supraspinatus posterior and both infraspinatus sub-regions in the scaption tasks (relative difference 47%-93%, p-values<0.0001–0.0079) (*e*). When using isometric best to normalize data, the %MVC were significantly greater compared to MEA 37% of the time (b) (p-values 0.0014–0.0067). No differences were detected between the isokinetic best and MEA methods (*a*). Graphical representation of the ER 30° fast experimental task comparing normalization methods by sub-region is shown in Figure 2.

**Table 4.** Comparison of means (sd) for each sub-region and task as normalized by different methods. Significant differences between normalization methods indicated with letters. a= significant difference between maximal electrical activity (MEA) and isokinetic best, b= significant difference between MEA and isometric best, c= significant difference between MEA and matched isokinetic, d= significant difference between isokinetic best and isometric best, e= significant difference between isokinetic best and matched isokinetic, and f= significant difference between isometric best and matched isokinetic. Alpha level was adjusted to 0.0083 (0.05/6) using Bonferroni correction. P-values are displayed in square brackets [].

Supraspinatus Anterior	MVC Normalization Method				
Experimental task	MEA	Isokinetic best	Isometric best	Matched isokinetic	Significance
ER30fast	14.11 (8.50)	17.39 (9.02)	20.04 (13.57)	29.47 (11.25)	*c [<0.0001], e [<0.0001], f [<0.0008]*
ER30slow	14.53 (8.91)	17.92 (9.28)	20.52 (13.37)	28.24 (7.37)	*c [<0.0001], e [0.0001], f [0.0033]*
ER90fast	14.41 (8.20)	17.77 (9.44)	20.65 (13.13)	29.52 (13.16)	*c [<0.0001], e [0.0005], f [0.0069]*
ER90slow	14.75 (8.65)	18.50 (9.90)	20.81 (14.39)	27.29 (12.40)	*c [<0.0001], e [0.0044]*
Scaption fast	18.02 (10.09)	21.48 (9.47)	25.74 (16.00)	27.37 (11.52)	*b [0.0063], c [0.0012]*
Scaption slow	17.80 (10.43)	21.56 (10.28)	20.04 (13.57)	26.71 (9.21)	*c [0.0007]*
Supraspinatus Posterior					
ER30fast	13.42 (10.34)	15.99 (11.00)	20.03 (12.48)	24.04 (10.45)	*b [0.0022], c [<0.0001], e [0.0003]*
ER30slow	13.95 (10.20)	16.56 (10.58)	20.78 (13.15)	26.44 (7.16)	*b [0.0050], c [<0.0001], e [<0.0001]*
ER90fast	14.03 (9.96)	16.54 (9.88)	21.99 (14.52)	27.13 (10.19)	*b [0.0042], c [<0.0001], e [0.0002]*
ER90slow	14.33 (10.87)	17.09 (11.00)	21.87 (16.27)	25.40 (10.33)	*c [0.0002], e [0.0039]*
Scaption fast	16.91 (10.83)	19.72 (11.25)	24.53 (14.97)	29.57 (14.48)	*c [0.0001], e [0.0018]*
Scaption slow	15.40 (9.85)	18.25 (10.69)	20.04 (12.48)	26.93 (16.95)	*c [0.0006], e [0.0079]*
Infraspinatus superior					
ER30fast	14.02 (9.93)	18.25 (11.46)	17.15 (11.89)	30.24 (12.38)	*c [<0.0001], e [<0.0001], f [<0.0001]*
ER30slow	13.68 (8.80)	17.83 (10.02)	16.28 (9.12)	31.45 (14.53)	*c [<0.0001], e [<0.0001], f [<0.0001]*
ER90fast	13.68 (9.25)	17.97 (10.49)	17.10 (11.80)	24.39 (10.27)	*c [<0.0001], e [0.0023], f [0.0006]*
ER90slow	13.54 (8.54)	17.86 (9.98)	16.43 (9.74)	25.83 (11.59)	*c [<0.0001], e [0.0009], f [0.0001]*
Scaption fast	12.00 (7.40)	15.18 (7.31)	15.18 (9.79)	24.23 (8.54)	*c [<0.0001], e [<0.0001], f [<0.0001]*
Scaption slow	10.93 (6.96)	14.21 (7.94)	17.15 (11.89)	22.38 (11.07)	*b [0.0066], c [<0.0001], e [0.0005]*
Infraspinatus middle					
ER30fast	12.15 (9.28)	16.53 (8.20)	21.11 (16.64)	26.35 (12.10)	*b [<0.0067], c [<0.0001], e [0.0032]*
ER30slow	12.99 (9.54)	17.40 (8.08)	22.22 (15.90)	25.33 (10.26)	*b [0.0039], c [0.0002]*
ER90fast	10.85 (8.53)	15.25 (8.10)	19.31 (14.96)	21.06 (9.73)	*b [0.0030], c [0.0005]*
ER90slow	14.55 (11.36)	17.73 (9.64)	22.03 (18.19)	25.13 (6.48)	*c [0.0025]*
Scaption fast	9.48 (7.27)	13.53 (7.41)	16.32 (12.72)	24.87 (10.98)	*c [<0.0001], e [0.0002], f [0.0038]*
Scaption slow	10.13 (8.27)	13.12 (7.45)	21.11 (16.64)	25.33 (10.83)	*b [0.0014], c [<0.0001], e [0.0005]*

## 4. Discussion

The aim of our study was to explore the differences in normalized data when using standard isometric MVCs compared to isokinetic MVCs for the sub-regions of supraspinatus and infraspinatus during dynamic tasks. Our hypothesis was that isokinetic MVCs would result in greater amplitude recordings for the majority of subregions and this proved to be true (35/64 individual subregions). There were no significant differences between normalized task data for the MEA and isokinetic best methods. In contrast, when using the isometric best method compared to MEA, there were significant differences seen 37% of the time with experimental task data normalized with the isometric best method having a higher %MVC. This suggests that typical isometric MVCs were not as effective at achieving high levels of activation for as many sub-regions as the isokinetic MVCs. These findings have implications for how %MVC data is interpreted for use in occupational and clinical settings.

Many different reference positions, both ER and abduction-based, produced high levels of electrical activity in the sub-regions of supraspinatus and infraspinatus. This is congruent with a previous study by Alenabi et al. that investigated different isometric MVC positions for activating the sub-regions of supraspinatus and infraspinatus for the purpose of normalizing EMG data (Alenabi et al. 2018). They found that no single test position maximally activated all sub-regions of these muscles. Instead, combinations of six to eight isometric MVC exertions were recommended to achieve greater than 90% activation in these sub-regions. The large variability in which exertions produce high activation for the muscles of the rotator cuff may relate to their role in shoulder stabilization (Alenabi et al. 2018). Previously, the posterior supraspinatus was more active with more arm elevation when performing an isometric ER task with maximal exertion (Alenabi et al. 2018). Similarly, our results show that the isokinetic ER MVC exertions, which involved either 30° or 90° of humeral elevation, resulted in more MEA occurrences than the isometric ER MVC where the arm was held by the side. Studies by Cudlip & Dickerson and Alenabi et al. also reported more anterior supraspinatus activation with increased humeral elevation (Alenabi et al. 2018; Cudlip and Dickerson 2018). In our study, the isokinetic MVCs may have been more effective for this sub-region as some degree of arm elevation was involved whereas the classic isometric MVCs were performed with the arm by the side. Abduction and scaption based MVCs also produced high activation of both regions of supraspinatus. When considered together, 75% of subjects had MEA during a scaption or abduction based MVC when compared to an ER based MVC for the anterior supraspinatus and 69% for the posterior supraspinatus. The opposite was true for the middle infraspinatus where ER MVCs were more effective with 69% of subjects achieving MEA.

In some cases, significant differences emerged by comparing the isokinetic matched to the isometric best and isokinetic best methods. For the supraspinatus sub-regions and the superior infraspinatus sub-region, when using the isokinetic matched method to normalize data from all ER tasks, the %MVC increased compared to the isokinetic best method by a relative degree of 47–69%. This can be attributed to the ER based MVCs generally producing lower electrical activity in these sub-regions; therefore the relative difference between the two methods was substantial. For the superior sub-region of infraspinatus the isometric MVCs achieved MEA more often than the isokinetic MVCs in contrast to the other sub-regions where the opposite was true. The isokinetic matched method of normalization resulted in higher %MVC in five out of six tasks for infraspinatus superior when compared to normalizing with isometric best with values for the same activity differing by a relative factor of 93%. As the isometric MVCs generally achieved higher electrical activity for the superior infraspinatus, the relative difference between the isometric best method and the isokinetic matched method was enhanced. The isokinetic ER MVCs in general may have represented a more novel movement pattern than the abduction or scaption MVCs. Subjects may therefore have felt more secure in an isometric position when performing the ER MVCs and were able to generate greater activation.

When using the isokinetic matched normalization method, significant differences when comparing to the MEA method of normalization occurred in all cases and relative %MVC values that were over 100% greater was common. Using the peak EMG from the task under investigation has been previously criticized as there is no guarantee that maximal activity is actually obtained and therefore presenting data as a %MVC and implying a certain degree of activation is inaccurate (Burden 2010; Halaki and Ginn 2012; Ball and Scurr 2013). Ideally, the MVC used to normalize data should maximally activate the target muscle so that representing data as %MVC gives meaning to the degree of muscle activation during various tasks. If the MVC exertion used fails to maximally activate the target muscle, %MVC values will be inflated making it difficult to compare relative activity between sub-regions impacting use of data in real-world applications. Clinically, the degree of muscle activation may be of interest to those who wish to obtain a high degree of tension in one region of the tendon, but not in another area which may be in a vulnerable state – for example a portion of the rotator cuff tendon with a tear or having undergone recent surgical repair. The most common complication following arthroscopic rotator cuff tendon repair is anatomical failure of the repair and as a result typical post-operative management involves a period of several weeks of immobilization to protect the susceptible tendon as it heals (Randelli et al. 2012; Sgroi and Cilenti 2018). This period of immobilization is then followed by progressive strengthening of the shoulder musculature (Sgroi and Cilenti 2018). During the strengthening phase, achieving sufficient activation in the undamaged tendon while unloading the vulnerable area may be desirable and thus having accurate information about the relative activity of sub-regions is critical for developing future exercise protocols that are safe and effective.

When comparing the isometric best MVC and MEA normalization methods for each sub-region and task, the isometric best MVC was significantly different 37% of the time and resulted in higher normalized %MVC values (Table 4 – (b)). The joint angle at which peak excitation occurs varies widely in shoulder musculature (Hodder and Keir 2013), and given a limited number of isometric positions were used, an individual may not have been at their optimal joint angle to produce their greatest electrical activity. Repeating multiple isometric MVCs at enough joint angles to capture the greatest electrical activity per participant would be time consuming and could still be unsuccessful compared to the practicality of using a dynamic MVC. Although dynamic MVCs performed at multiple speeds without the constraint of constant velocity, for example using the resistance of a researcher and a metronome, was not examined, it may represent a more accessible means to produce dynamic MVCs.

## 5. Limitations

A number of limitations within this study should be noted including the exclusion of the inferior infraspinatus sub-region due to poor quality and inconsistent signal. Investigating the inferior infraspinatus in this manner is still quite novel and the point of insertion likely didn’t position the electrode in the muscle optimally for signal pick-up and modifying electrode placement protocols may produce better results. Additionally, given the length of the protocol a limited number of isokinetic and isometric test positions could be tested. Not all laboratories are equipped with a dynamometer which is also a limiting factor for the general applicability and practicality of using isokinetic MVCs.

## 6. Conclusions

This study compared isokinetic and isometric MVCs to normalize data from supraspinatus and infraspinatus sub-regions during a dynamic task. Isokinetic MVCs produced greater amplitudes than isometric MVCs, and using isometric MVCs to normalize task data resulted in higher normalized task %MVC levels compared to normalizing to the maximum electrical activity elicited. There were no significant differences when normalizing using isokinetic MVCs compared to the maximal electrical activity method. Several exertions are capable of bringing about high electrical activity in the sub-regions of supraspinatus and infraspinatus with both isometric and isokinetic exertions. Given both ER and abduction-based positions produced maximums in each sub-region, each should be included when selecting MVC positions for these sub-regions and positions should involve varying degrees of arm elevation. If using isometric MVCs, then ensuring a sufficient number of positions are used is imperative and using isokinetic MVCs may represent a more efficient means of achieving greater electrical activity across all participants. The fatigue responses to a task are often characterized in terms of the distribution of %MVC muscle demand during a task, and thus obtaining as close to a true maximal activation of the sub-region in question during MVC collection is critical for data interpretation. For this reason, use of dynamic MVCs should be considered with future research.

## Data Availability

The data that support the findings of this study are available from the corresponding author, S.K., upon reasonable request. https://authorservices.taylorandfrancis.com/data-sharing/share-your-data/data-availability-statements/
